# AI-Powered Visual Sensors and Sensing: Where We Are and Where We Are Going

**DOI:** 10.3390/s25061758

**Published:** 2025-03-12

**Authors:** Hieu Nguyen, Minh Vo, John Hyatt, Zhaoyang Wang

**Affiliations:** 1 School of Electrical Engineering, International University, Ho Chi Minh City 700000, Vietnam; nthieu@hcmiu.edu.vn; 2 Vietnam National University, Ho Chi Minh City 700000, Vietnam; 3 Neuroimaging Research Branch, National Institute on Drug Abuse, National Institutes of Health, Baltimore, MD 21224, USA; 4 SpreeAI, Incline Village, NV 89450, USA; minh.vo@spreeai.com; 5 U.S. Army Research Laboratory, 2201 Aberdeen Boulevard, Aberdeen, MD 21005, USA; john.s.hyatt11.civ@army.mil; 6 Department of Mechanical Engineering, School of Engineering, The Catholic University of America, Washington, DC 20064, USA

## 1. Introduction

Deep learning, a machine learning method that mimics the neural network structures of the human brain to process data, recognize patterns, and make decisions, traces its origins back to the 1950s. It was not until the beginning of the 21st century that deep learning truly began to flourish, with the breakthrough in algorithms, the significant increase in computing power, and the advent of large-scale data acquisition. As a subset of artificial intelligence (AI), deep learning has acted as a driving force behind strengthening the impact of AI in different fields and enhancing its integration into daily life.

In 2006, Geoffrey Hinton and his student [1] showed that deep belief networks, stacks of restricted Boltzmann machines, could be trained layer by layer in an unsupervised manner and fine-tuned using supervised learning. This model addressed the vanishing gradient problem and allowed for the practical training of multilayer neural networks. It laid the foundation for subsequent advancements and marked the revival of deep learning. A landmark success came in 2012 when AlexNet [2] delivered groundbreaking results on images from the ImageNet Large Scale Visual Recognition Challenge (ILSVRC), outperforming all previous methods. This achievement highlighted the overwhelming capabilities of deep convolutional neural networks (CNNs) and quickly attracted widespread attention from both academia and industry.

Over the past decade, deep learning has undergone a quick growth and has driven numerous breakthroughs in AI. Some notable early achievements include, but are not limited to, the dropout scheme [3] introduced in 2013, recurrent neural networks (RNNs) [4,5] and generative adversarial networks (GANs) [6] proposed in 2014, GoogleNet [7] and residual neural network (ResNet) [8] in 2015, and DeepMind’s WaveNet model [9] and AlphaGo model [10] in 2016. It should be noted that some of the papers were introduced as preprints in one year but were formally published in a subsequent year, so there may be a difference between the year of introduction and the year of publication. The year 2016 marked the successful transition of deep learning from theoretical research to practical applications, and more sophisticated models have since started to blossom.

Google’s Transformer architecture [11], introduced in 2017, abandoned traditional RNNs or CNNs in favor of a self-attention mechanism, substantially improving the performance of sequence modeling tasks, such as natural language processing (NLP). Google’s BERT model [12], introduced in 2018, and OpenAI’s GPT-3 model [13], introduced in 2019, both demonstrated powerful text generation and comprehension capabilities. They further advanced the application of large models in NLP. Between 2020 and 2021, a Google team proposed the Vision Transformer (ViT) [14], successfully applying Transformers to computer vision tasks and challenging the dominance of convolutional neural networks in image recognition. The release of YOLOv4 [15] demonstrated the efficiency and accuracy of deep learning in real-time image analysis. Additionally, GANs and their variants, such as BigGAN [16] and StyleGAN2 [17], continued to improve performance in image and video generation tasks. In 2022, the launch of ChatGPT [18] (developed by OpenAI in San Francisco, CA, USA) created a global sensation, marking a major breakthrough in human–computer interaction and dialogue systems and setting new standards for future intelligent assistants and AI customer service. OpenAI also introduced several large-scale models based on Transformers, such as DALL·E, which generates images from textual descriptions, the cross-modal model CLIP, and the code generation model Codex, all of which obtained considerable attention in their respective fields. In 2023, Meta (Menlo Park, CA, USA) released Llama [19], a highly efficient and accessible family of language models. It aims to achieve high performance with fewer computational resources to facilitate advanced AI research and applications. Llama’s emphasis on accessibility and open collaboration may significantly affect the trajectory of AI development. In late 2023, not surprisingly, Google (Mountain View, CA, USA) launched Gemini [20] as a competitor to ChatGPT. Then, in 2024, OpenAI introduced Sora, a cutting-edge tool to convert text into video [21]. This technological breakthrough generated widespread excitement and amazement around the world. Amid the ongoing astonishment, Sora quickly faced intense competition from emerging tools such as Google’s Veo 2 (developed by Google DeepMind, Mountain View, California, USA), Kuaishou’s Kling (developed by Kuaishou, Beijing, China), Runway (developed by Runway AI, New York, USA), etc. This speedy development indicates how technology is advancing at an unprecedented pace in the AI era.

The aforementioned milestone events have tremendously influenced academia and driven technological innovation and application across industries. For instance, companies in the autonomous driving sector, like Tesla and Waymo, rely on deep learning algorithms to enhance vehicle perception and decision-making [22,23]. In healthcare, deep learning models are employed to analyze medical images and assist doctors in diagnosing diseases [24]. With the ongoing technological evolution, the future of deep learning promises broader applications and more exciting innovations and discoveries. It will continue to fundamentally change how we live and work. It is particularly noteworthy that both the 2024 Nobel Prize in Physics and the 2024 Nobel Prize in Chemistry were awarded for research work related to deep learning.

As deep learning and AI pervade nearly every field of engineering and science, computer vision remains one of the key areas of application, which has been considerably enhanced and expanded. Integrating AI with computer vision-based sensors and sensing technologies has resulted in many groundbreaking advances, such as highly accurate object detection [25], facial recognition [26], image segmentation [27], optical character recognition [28], human pose estimation [29], and real-time 3D reconstruction [30,31]. These are challenging to achieve with conventional methods due to the combined accuracy, speed, simplicity, and efficiency required.

In 2022, we launched a Special Issue on the progress of AI in computer vision research and applications, which focused on new vision-based sensors and measurement technologies. The Special Issue featured 30 articles covering a wide range of methods and applications. This editorial aims to briefly summarize these articles, along with providing insights into the future development of related technologies. Furthermore, recognizing the rapid advancement of technologies in this field, we launched a new edition of the Special Issue to present the latest progress and innovations in AI-powered computer vision for sensors, sensing, and measurement research, along with their applications in engineering.

## 2. Where We Are

The previous Special Issue published 30 high-quality articles spanning multiple fields related to sensors and sensing technologies. Here, we provide a brief review and summary of these contributions.

### 2.1. Depth Estimation and 3D Reconstruction

The field of 3D reconstruction and depth estimation is rapidly evolving, with innovative techniques leveraging deep learning and advanced algorithms to enhance accuracy, efficiency, and real-time performance across various applications. Several recent contributions that significantly advance this domain are summarized below.

Ibrahem and colleagues (Contribution 1) presented a novel method for dynamic depth estimation via Vision Transformers (ViTs) in the encoder branch. By introducing different versions of ViTs, ViT-t16, and ViT-s16, the technique effectively manages the computational demands typical of ViTs. This optimization supports the balance between speed and accuracy and allows the system to operate at approximately 17 frames per second, which makes it suitable for dynamic applications.

Liu’s team (Contribution 2) proposed a deep learning-based stereo-matching framework, ASR-Net, which integrates a feature extractor network with a residual depth refinement. The depth estimation process can gradually start refining at low resolution and follow a hierarchical strategy to improve the depth accuracy by using novel deformable convolution techniques and adaptive cost aggregation. This method has shown remarkable improvements in both speed and accuracy across several datasets, such as kitti2015, kitti2012, and scene flow.

Considering the accuracy and speed balance in the 3D Morphing Model, You et al. (Contribution 3) proposed a lightweight network to improve the 3D deformation model, called Mobile-FaceRNet. The training network leverages the multi-scale representation approaches and separable convolutions, together with novel perceptual loss and unique residual attention modules, to produce high-fidelity facial reconstructions that remain robust against variations in pose and obstructions.

Wang and co-authors (Contribution 4) presented a deep learning-based 3D shape measurement method built on the traditional fringe projection profilometry (FPP) technique. The technique uses invisible fringe patterns in near-infrared spectra and a CNN-based framework, which adopts a 3D collaborative filtering and block-matching approach. Compared to previous integrated and traditional denoising techniques, the proposed technique improves the 3D shape measurement accuracy using a near-infrared system and increases the computational speed using the learning-based method.

An article by Wang et al. (Contribution 5) involved the use of a residual graph CNN for the 3D reconstruction of the left myocardium with cardiac magnetic resonance (MR) images. This method combines initial mesh generation, vertex joint feature learning, and mesh deformation. The initial mesh is reconstructed via segmentation and the Cubify algorithm, the deformed mesh is iteratively constructed via residual graph CNN, and the skeleton of vertices at different receptive fields is obtained using an autoencoder neural network. The results were obtained with cardiac MR images and show the enhanced accuracy and robustness of the reconstruction process compared to existing methods.

Felipe and colleagues (Contribution 6) highlighted how errors in the pitch angle can lead to significant distortion in 3D scene reconstruction. They proposed a machine learning-based approach relying on regression algorithms to estimate and correct these pitch angle errors. They used a range of regression methods, including Linear Regression, Regression Trees, Regression Forests, and Multi-Layer Perceptron, trained on a variety of input–output pairs that capture different real-world situations. This helped the calibration process reduce distortion, resulting in more accurate 3D scene reconstructions.

### 2.2. Segmentation and Object Detection

Deep learning plays a vital role in image segmentation and object detection applications. The corresponding methods adopt complex algorithms and neural network architectures to improve accuracy and reliability, increase the processing speed, and address the complexities associated with various data conditions. The following contributions highlight the latest developments in this field.

Recognizing the importance of the sustainable development of intelligent fisheries, Han et al. (Contribution 7) presented a variation of the PSPNet network, known as IST-PSPNet, designed for fish segmentation. The method features an iterative attention feature fusion mechanism for capturing detailed features across multiple scales. It also utilizes a SoftPool pooling technique to reduce parameters and computational load while retaining important feature information. A triadic attention mechanism, called triplet attention (TA), enhances the focus on specific fish body characteristics. The method demonstrated its effectiveness on the DeepFish dataset, yielding a mean Intersection over Union (mIoU) of 91.56%, with 46.68 million parameters and 40.27 GFLOPS.

Dang et al. (Contribution 8) introduced a variation of the semantic segmentation network incorporating quantization techniques to extract corridor scenes from a single image, facilitating robot navigation. The architecture includes autoencoder MobilenetV2 with multi-scale fusion characteristics, enhancing segmentation accuracy while minimizing computational demands. A new balanced cross-entropy loss function was introduced, and the network model was trained using four datasets: CitySpaces (5000 images), KITTI (400 images), Duckie-dataset from Ducktown (1200 images), and TaQuangBuu’s dataset (1200 images).

Recognizing the limitations of multi-scale receptive fields in a real-time using segmentation network, MFAFNet (Contribution 9) was proposed to tackle this with a parallel structure to extract short- and long-range contextual information and a separable asymmetric reinforcement non-bottleneck module to preserve spatial details. The network was tested on Cityscapes and CamVid; it achieved 75.9% and 69.9% mean IoU with 60.1 and 82.6 FPS, using only 1.27 million parameters.

Regardless of the use of a multi-scale feature fusion module, Su’s work (Contribution 10) proposed an instance segmentation method for the rubber ball cleaning system. Unlike previous backbone networks with the convolution module, this study employed a Pyramid Vision Transformer with the attention mechanism to enhance the feature extraction and reduce the computational cost. In addition, they improved the feature fusion module across the scales to enhance the output feature representation. Compared to DeepMask, Mask R-CNN, BlendMask, SOLOv1, and SOLOv2, their model improved the Dice score, Jaccard coefficient, and mAP by 4.5%, 4.7%, and 7.73%, respectively, achieving 33.6 FPS and 79.3% segmentation accuracy.

Anomaly detection and segmentation techniques are increasingly being used in areas such as manufacturing quality control and fraud detection. In their research (Contribution 11), Candido de Oliveira and his team introduced a unique loss function designed for training a deep convolutional autoencoder, utilizing only images of unaltered boards. This loss function focuses on analyzing higher-level features to compare the original image with the autoencoder’s output, which enhances the ability to segment various structures and components. They validated their approach through experiments using a dataset that mimics real-world conditions, and they claimed that their model outperformed other leading techniques in the field of anomaly segmentation for the tested scenarios.

In their research (Contribution 12), the authors introduced a streamlined network segmentation model named SEMD, which aimed to precisely segment images of standing trees against complex backgrounds. This model utilizes multi-scale fusion with DeepLabV3+ to reduce the loss of feature information and incorporates the MobileNet architecture to enhance computational efficiency. Additionally, an attention mechanism known as SENet is included to effectively capture essential features while filtering out irrelevant data. The experimental results indicate that the SEMD model achieves a Mean Intersection over Union (MIoU) of 91.78% in simpler settings and 86.90% against more intricate backgrounds.

Acknowledging the limitations of prior deep learning methods, for instance, segmentation, (Contribution 13) presented a new technique called Boundary Refine (BRefine), which enhances segmentation quality and detail. This method leverages the FCN backbone for segmentation, along with a multistage fusion mask head to boost mask resolution. It also introduced BRank and sort loss (BR and S loss) to tackle segmentation inconsistencies and improve boundary detection. In comparison with previous models like Mask R-CNN, BRefine showed improvements of 3.0, 4.2, and 3.5 AP on the COCO, LVIS, and Cityscapes datasets, respectively, with a further enhancement of 5.0 AP for large objects within the COCO dataset.

The research documented in (Contribution 14) combined YOLOv5s for object detection with Deeplabv3+ for image segmentation to facilitate meter-reading extraction. The YOLOv5s model first localizes the meter dial, followed by Deeplabv3+, which uses a MobileNetv2 backbone to effectively extract tick marks and pointers. The results demonstrated that this methodology enables the YOLOv5s model to achieve an impressive mean average precision of 99.58% (mAP50) on the dataset, along with a rapid detection speed of 22.2 ms.

The detection transformer (DETR), which utilizes a Transformer-based framework for object detection, has attracted significant interest due to its strong performance on the COCO val2017 dataset. However, these models face challenges when applied to new environments that lack labeled data. To tackle this issue, (Contribution 15) proposed an unsupervised domain adaptive technique known as DINO with cascading alignment (CA-DINO). The approach introduces attention-enhanced double discriminators (AEDD) and weak category-level token restraints (WROT). AEDD aligns with the local and global contexts, while WROT extends the Deep CORAL loss to adjust class tokens after embedding. Experimental results on two rigorous benchmarks indicated a 41% relative performance boost compared to the baseline on the Foggy Cityscapes dataset.

Deep learning techniques have also been employed to detect liquid contents. The method outlined in (Contribution 16) focuses on identifying liquids within transparent containers, which is beneficial for various specialized applications, including service robots, pouring robots, security inspections, and industrial monitoring.

Rather than depending on conventional object detection techniques that utilize visible imaging, the research in (Contribution 17) approaches the challenge of accurately detecting weak infrared targets in complex environments while fulfilling the real-time detection needs. The authors developed a Bottleneck Transformer architecture and implemented CoordConv techniques to enhance detection performance. This methodology led to a notable accuracy increase, achieving a mean Average Precision (mAP) of 96.7%, which reflects a 2.2 percentage point improvement over Yolov5s, outperforming other leading detection algorithms.

In the realm of human pose estimation, heatmap-based strategies have been predominant, offering a high performance but facing difficulties in accurately detecting smaller individuals. To remedy this, SSA Net (Contribution 18) proposes an innovative solution. It employs HRNetW48 as a feature-extractor and utilizes the TDAA module to bolster the perception of smaller scales. SSA Net replaces traditional heatmap methods with coordinate vector regression, attaining an impressive AP of 77.4% on the COCO Validation and competitive scores on the Tiny Validation and MPII datasets, showcasing its capability across different benchmarks.

### 2.3. Literature Review

Object detection plays a crucial role in computer vision, significantly enhancing situational awareness and surveillance within marine environments. The research presented in (Contribution 19) examines various models designed to bolster maritime surveillance systems, with an emphasis on ship localization, classification, and detection. In a related domain, deepfake technology (Contribution 20), which creates counterfeit audio, images, and videos, raises significant concerns regarding privacy, democracy, and national security. Consequently, considerable efforts have been directed toward developing methods to identify digital manipulations. Contribution 21 in the Special Issue introduced a novel approach to identifying the face images generated by deep networks, utilizing different color spaces. This network evaluates the variations in the color space components in a deep learning framework, enhancing face sensitivity and the model’s discriminative capabilities through the implementation of a channel attention mechanism.

Moreover, (Contribution 22) explored the concept of counterfactual fairness in the classification of facial attributes. The method employs a causal graph-based technique for attribute translation, generating realistic counterfactual images while taking into account the intricate causal relationships among various attributes through an encoder–decoder model. A causal graph is used to sample both factual and counterfactual facial attributes from a chosen face image. Extensive experiments conducted on the CelebA dataset showcase the proposed method’s effectiveness and interpretability in the realm of multi-attribute facial classification.

### 2.4. Applications

Addressing mismatches in computer vision—especially during the matching of image pairs—is essential due to the inherent geometric and radiometric disparities that often exist between images. These discrepancies can undermine the reliability of matching results, consequently impacting the accuracy of various vision-related tasks. Recognizing the limitations of supervised learning techniques and the challenges in accurate labeling, the authors of Contribution 23 introduced an innovative method that employs deep reinforcement learning (DRL). They developed an unsupervised learning framework named Unsupervised Learning for Mismatch Removal (ULMR). When compared to traditional supervised and unsupervised learning methods as well as conventional handcrafted techniques, ULMR shows enhanced precision, a higher retention of correct matches, and a decrease in false matches.

In the realm of video surveillance and behavior recognition, deep learning has showcased its potential, particularly in the medical sector. The approach detailed in Contribution 24 offers a comprehensive system for behavior-based video summarization and visualization, which aimed to monitor and evaluate the health and well-being of dogs. This system consists of multiple phases, such as video acquisition and preprocessing, object detection and cropping, dog behavior detection, and the creation of visual summaries that illustrate the dog’s location and behavioral patterns.

Another significant application of deep learning in video monitoring can be found in sewer maintenance and cleaning operations. The project described in Contribution 25 introduced the S-BIRD (Sewer-Blockages Imagery Recognition Dataset) to raise awareness about the prevalent issue of sewer blockages caused by materials like grease, plastic, and tree roots.

In the context of video monitoring, the challenge of identifying threats in X-ray baggage with a scarcity of labeled data is tackled by the FSVM model, a few-shot SVM-constrained approach outlined in Contribution 26. FSVM incorporates a differentiable SVM layer to enhance decision propagation and employs a combined loss function that includes SVM loss. Evaluation using the SIXray dataset indicates that FSVM surpasses four widely-used few-shot detection models, especially in complex dataset scenarios.

### 2.5. Computational Efficiency Optimization

The challenge of the high memory usage and computational demands in complex neural networks, such as VGG, is significant. The method introduced in Contribution 27 tackles this problem by automatically pinpointing convolutional channels that remain inactive throughout the training phase. This is achieved through two innovative loss functions: channel loss and xor loss. By adopting this strategy, notable enhancements were observed, including a increase in the speed of image generation of up to 49% and a 20% reduction in the number of parameters, all while preserving effective style transfer performance on the CIFAR-10 dataset.

The research in Contribution 28 uses a variational optical flow model to create a subgrid-scale optimization technique to accurately model intricate fluid motions within image sequences and calculate their two-dimensional velocity fields. By merging aspects of incompressible fluid dynamics with large-eddy simulation techniques, this approach effectively divides motion into both large-scale and small-scale turbulence elements, utilizing the Smagorinsky model. The newly developed subgrid scale Horn–Schunck (SGS-HS) optical flow algorithm demonstrated enhanced performance compared to conventional methods like Farneback dense optical flow, particularly in turbulent situations. It incorporated a velocity gradient constraint to boost accuracy in open-channel flow velocimetry experiments.

The investigation presented in (Contribution 29) confronts the obstacles of real-time object detection by suggesting a hybrid hardware–software framework. This framework aims to optimize computer vision algorithms across a variety of platforms, from smartphones to surveillance systems, with a particular focus on improving memory bandwidth efficiency and minimizing energy consumption. The research delves into the allocation of algorithm components to hardware resources, such as IP Cores, and elaborates on methods to facilitate interaction between hardware and software. Utilizing embedded AI, this approach dynamically configures and manages hardware resources, which enhances performance and adaptability in object detection tasks. The experimental findings illustrated the substantial advantages of employing IP Cores within an FPGA-based system, demonstrating noticeable improvements in the overall effectiveness and efficiency of the algorithms.

In Contribution 30, an advanced framework known as Multi-Modality Adaptive Feature Fusion (MMAFF) was proposed for skeleton-based action recognition, utilizing graph convolutional networks. This framework introduces multi-scale adaptive convolution kernels and various dilation rates, significantly improving the network’s capacity to accommodate diverse receptive fields across multiple layers and datasets. A self-attention mechanism was incorporated to refine traditional multi-scale temporal convolution, allowing for the adaptive selection of convolution parameters. Moreover, the research addressed the challenges related to context aggregation and initial feature fusion through a novel feature-fusion mechanism that replaces conventional residual connections. MMAFF exhibited competitive results on established benchmark datasets such as NTU-RGB+D 60 and NTU-RGB+D 120, highlighting its effectiveness in extracting spatial and temporal features for multi-modal action recognition tasks.

## 3. Where We Are Going

Given the pivotal role of 3D visual sensing and depth perception in the highly competitive and investment-intensive field of autonomous driving, achieving real-time, high-accuracy 3D imaging and depth measurement with comprehensive whole-field coverage remains one of the most actively researched technologies in computer vision and artificial intelligence. The approaches can be classified into two primary categories: the first directly extracts depth and 3D information from real-time video stream captured by single or multiple cameras using AI algorithms; the second utilizes AI to match the full-field pixel points of images captured by two (or multiple) cameras, and then employs conventional algorithms to calculate the depth and 3D point cloud data. The first approach is straight and aligns with the desired goal, while the latter may offer higher reliability through its stepwise process. Although deep learning-based 3D imaging and depth perception technologies, along with related software and hardware advancements (such as coping with interference from fog, rain, and snow [32,33]), are progressing rapidly, a general AI model capable of adapting to diverse real-world scenarios remains lacking. Nevertheless, we are optimistic that significant breakthroughs will come soon.

In addition to AI-powered 3D imaging and depth perception, AI and deep learning can enhance many other established sensing techniques.

### 3.1. Enhancing Established Methods with Deep Learning

In scientific research and engineering applications, we sometimes face challenges where we do not have access to certain techniques to carry out specific sensing or measurement tasks for various reasons or the available conventional methods fail to provide expected results. In such instances, the use and integration of AI can help solve the problem. Three illustrative examples that we recently obtained in preliminary research are presented in the following.

**Figure 1:**
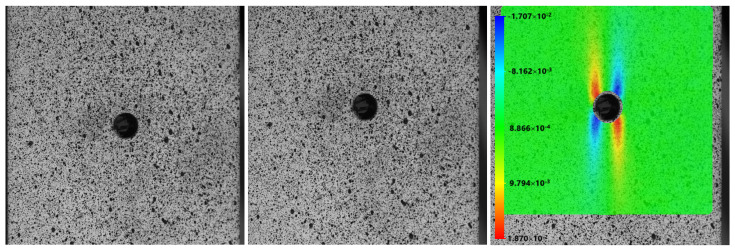
Strain determination of a thin plate with a hole under tensile loading using a deep learning scheme. From left to right: the initial shape, the deformed shape, and the corresponding shear strain map.

In structural and material characterization testing, it is often necessary to measure the full-field strain distribution of a target object. Digital image correlation (DIC) is currently a popular technique for such testing. However, the complexity of DIC algorithms has limited their implementation to a small number of companies and research teams for general and practical applications. In addition, the accuracy of the DIC results in advanced measurements often depends on various analytical control parameters, which require the user to have solid expertise in the technique. Using deep learning methods, accurate full-field strain measurements can now easily be achieved through a black-box-like approach, as shown in Figure 1. This example features a classic uniaxial tensile test on a thin plate with a circular hole in the center, where the stress concentration is well-documented at the edge of the hole. Unlike conventional methods, the deep learning-based approach is much more straightforward, without the need to specify numerous analysis control parameters. This preliminary exploration work uses a CNN encoder–decoder architecture, and the training was performed using a simulated dataset generated from theoretical solutions of classical solid mechanics problems [34,35,36]. The DIC technique is currently one of the most, if not the most, widely adopted methods in the field of experimental mechanics, and we can expect that AI-powered techniques may soon replace the existing algorithms of the DIC technique due to their considerable advantages.

**Figure 2:**
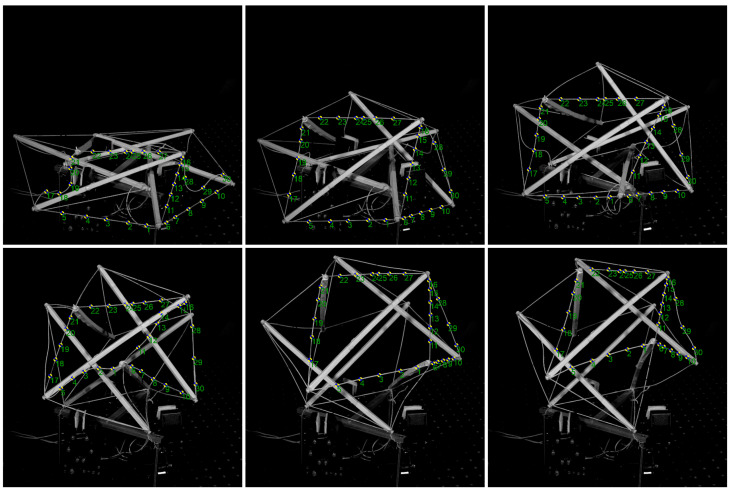
AI−powered dynamic motion tracking of points in a tensegrity structure. Unlike conventional methods, the deep learning-based approach can accurately track all points of interest. The images were captured at 6600 fps, and the frame interval of the six representative images shown here is 200.

In the second example, we show the dynamic motion measurement of a tensegrity structure, as illustrated in Figure 2. This experiment faces two notable challenges: first, the points of interest are small and highly similar, making them prone to misidentification; second, occlusion and overlap hinder the conventional analysis from accurately tracking these points, leading to measurement errors. Deep learning techniques, however, can effectively address these issues and accurately track each point, much like the cognitive capabilities of the human brain. The model in this preliminary work uses a CNN and correlation transformer architecture [37].

**Figure 3:**
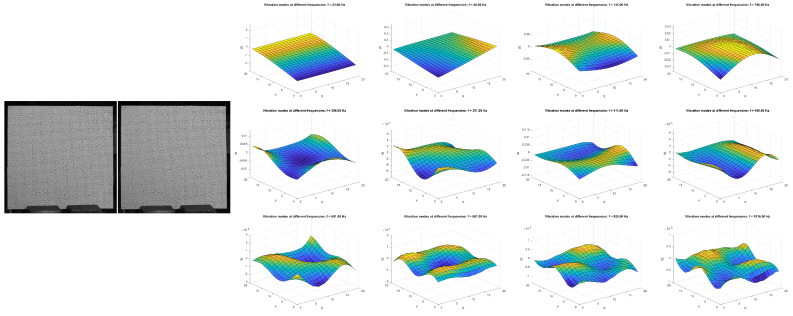
AI−powered analysis of free vibration modes in a thin plate. The images were captured at a high speed of 14,000 fps. Presented are two typical frames along with 12 identified vibration modes.

The third example demonstrates the ability of a deep learning approach to identify mechanical vibration modes (Figure 3). In this pilot study, multiple vibration modes, specifically, amplitude and phase information for each point within the region of interest, were extracted from a high-speed video clip of a freely vibrating thin plate. To prepare the training dataset, theoretical solutions [38] were applied to the same plate (with one side fixed and the other three sides free) to generate simulated video frames. The preliminary work utilizes a CNN and transformer architecture.

The above three examples demonstrate that AI-powered visual sensors and sensing have opened up exciting possibilities for unprecedented performance in numerous scientific and engineering applications. However, there are several challenges that researchers and engineers must address in order to fully realize the potential of these AI-powered technologies. One of the primary challenges is the difficulty of capturing real-world datasets. Visual sensors rely on large amounts of data to train deep learning models effectively, but acquiring high-quality, diverse, and representative datasets can be time-consuming and expensive, and often requires complex equipment. Moreover, in certain environments, such as extreme conditions or hard-to-reach locations, gathering the necessary data may not be feasible.

A natural way to deal with the real dataset preparation issue is to use theoretical simulation datasets, as shown in the examples mentioned previously. Simulated datasets, though less resource-intensive to generate, often struggle to capture the nuanced complexities of real-world scenarios. For example, complex geometries and deformation fields are difficult to rigorously simulate. As a result, AI models trained on such datasets may struggle to generalize to new and unseen environments in real-world applications.

Additionally, there is no one-size-fits-all deep learning model for general sensing applications, and identifying or designing the right model, that performs well across a wide range of tasks, remains a major obstacle. Researchers often need to customize or fine-tune models based on complex trials, which can lead to long research and development cycles.

Looking toward the future, several promising directions could help unlock the full potential of AI-powered visual sensors. One such approach is the use of finite element simulation to generate highly detailed synthetic datasets that simulate various physical problems. By combining FEM with AI techniques, it is possible to create datasets that reflect a wide range of scenario factors, such as stress, deformation, temperature, velocity, and electric and magnetic field intensities, which would be difficult or expensive to capture in the real world.

Dataset preparation may also utilize game engines like Unreal Engine and Unity [39] to generate realistic and controlled datasets. These engines are capable of simulating diverse environments with high accuracy. They offer flexibility in manipulating variables such as lighting, object positioning, and texture (e.g., transparent and high-reflective objects, which are typically hard to measure for many sensors), making them an invaluable tool for testing and training visual sensors under a broad range of situations.

It is important to distinguish the aforementioned simulated data from the “synthetic data” produced via generative AI, which often cannot safely be used to train another AI model [40].

**Figure 4:**
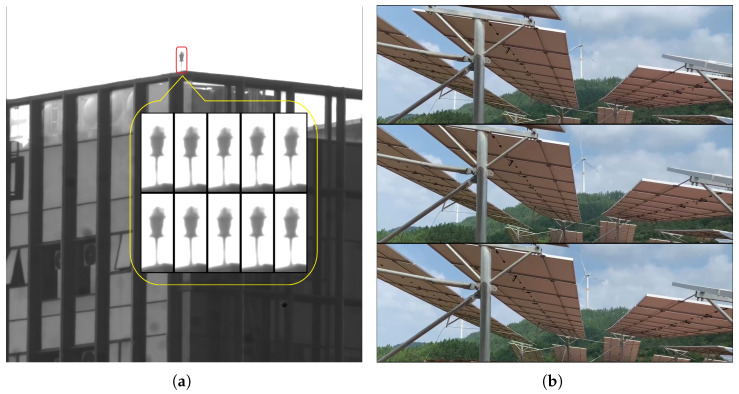
AI should be capable of distinguishing disturbances from the physical quantities that need to be measured. (**a**) Image distortion in long-range sensing caused by variations in air density and thermal haze. Ten representative frames of a target are zoomed-in for better visualization; (**b**) The motions of the solar panels under wind force, as captured by an outdoor camera, include the rigid-body motions of the camera itself.

In recent years, one of the fastest-growing engineering applications of vision-based sensing is non-contact movement detection, deformation measurement, and the health monitoring of structures over long distances. The biggest challenge in accurately extracting the desired information lies in eliminating visual distortions caused by variations in air density and thermal haze over long-range measurements, as shown in Figure 4a. These image distortions introduced by atmospheric effects can severely compromise the accuracy of motion and deformation analysis and often lead to errors in data interpretation. As AI-powered sensing systems are increasingly being integrated into key engineering applications such as remote sensing, autonomous vehicles, and surveillance, eliminating interference is critical to obtaining reliable input image data. This is a technically demanding task with a high priority in the field.

While some progress has been made to reduce atmospheric distortion through hardware and software solutions [41], much work remains. Future advancements in AI-powered computer vision should include exploring algorithms capable of isolating and compensating for such distortions. Solving this problem will not only improve the accuracy and reliability of long-range motion and deformation measurements but also unlock new levels of performance for broader applications. For instance, Figure 4b shows an application where the images documented both the vibrations of the solar panels and the more significant movements of the outdoor camera. To accurately characterize the vibrations of the panels under wind conditions, it is crucial to isolate the effects of the camera’s motion.

**Figure 5:**
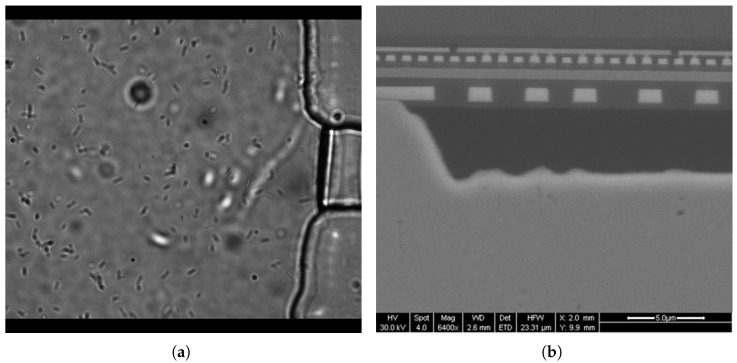
Visual sensing for motion and strain measurements using microscopy. (**a**) Bacteria motion tracking under an optical microscope. It is difficult to accurately track all the bacteria motions; (**b**) Microelectronics strain measurement under a scanning electron microscope. It is difficult to determine the full-field strain map.

Figure 5 shows two microscopy measurements we encountered in the research. The measurements not only highlight the applications of visual sensing technology in fields such as biology and microelectronics but also demonstrate the difficulties of conducting them using conventional methods. We believe that AI-powered techniques, as previously described, can provide convenient and effective solutions to these challenges.

Most existing AI-powered visual sensing techniques adopt network architectures, including CNNs, RNNs, and GANs. A current and future approach is the use of transformer architectures in deep learning. Transformers, known for handling sequential data and long-range dependencies, have obtained impressive results in natural language processing and computer vision tasks [42]. They have the potential to enhance sensing and measurement performance in every aspect by correlating both local and global features in the visual data, in addition to other advantages, such as scalability to larger datasets and adaptability to different input types. How to reduce the number of model parameters and lower the computing cost while maintaining performance will also be a research topic of interest.

### 3.2. Moving Beyond Conventional Deep Learning

Due to the enormous amount of investment in AI, existing deep learning methods are constantly being adapted to new problems and incrementally improved (including the release of new and more powerful models). The adoption of AI continues to accelerate as a result. At the same time, the current methods are subject to some fundamental constraints. For example, current state-of-the-art models rely on universal function approximation theorems [43,44], resulting in models with tens of billions of parameters and power demands showing as much as a 100% increase in global data center power consumption in the last 5 years [45]. Likewise, the existing methods are predominantly correlative, leading to limited generalization and causal reasoning, etc. The emerging research increasingly aims to address these limitations.

The current state of the art in AI lacks a general framework to develop model architectures whose inductive bias is appropriate for processing arbitrary data types, although a handful of specific model archetypes have been identified for particular purposes. For example, CNNs are orders of magnitude more parameter-efficient than fully connected networks for modeling data types whose features are translationally invariant (e.g., images); likewise, attention-based methods such as transformers are suitable for sequence-based data. In many cases, models can be successfully applied to data types whose features do not match their inductive bias. For example, vision transformers convert image patches to sequences via rastering, but the resulting models are extremely large, computationally expensive black boxes.

These challenges are exacerbated for multimodal data. In order to make predictions based on heterogeneous data types, model inputs must be embedded into a common space, typically via modality-specific encoding layers. This is particularly effective for modes with similar feature spaces (e.g., RGB and IR). Beyond these cases, AI-powered sensing has the potential to integrate data from very heterogeneous modes, views, and resolutions to decrease uncertainty and improve predictive power. Examples might include combining data from acoustic sensors and cameras or from multiple cameras with non-overlapping fields of view. How to realize these abilities without prohibitively increasing model size and operational cost remains an open question.

One promising cost-reduction approach that can be inserted into existing data–model pipelines is to incorporate an extremely lightweight, near-sensor model that only determines whether a feature of interest may be present. The data are passed to the downstream large model for full, costly processing only if this feature is detected [46]. In addition, several fundamentally new approaches to AI modeling have seen rapid growth over the past few years, although, in addition to open research questions, hardware suitable for these methods is not as mature as that for conventional deep learning.

Hyperdimensional computing [47] encodes data into high-dimensional, nearly orthogonal vector representations, which can be processed with a small library of simple vector-algebraic operations. The benefits of this approach include the comparatively transparent “reasoning” regarding the data, high robustness to data faults, a low computational cost, rapid training, and the straightforward integration of multimodal data [48]. Neuro-symbolic AI combines conventional deep learning for feature extraction with symbolic logic for transparent, robust causal reasoning, and generalizability [49]. In particular, conventional deep learning is purely correlative by construction, and models capable of causal reasoning represent a significant advance.

## 4. Closing Remarks

Recently, OpenAI announced the o3 model, which has significantly improved reasoning and problem-solving capabilities compared to previous models. The exceptional performance of the experimental model on science and math tests has surprised researchers [50]. Meanwhile, a new startup, DeepSeek, revealed that the cost of the computing power required to train a large model can be a fraction of that demanded by existing models for similar tasks [51], which has already spurred big tech corporations to reexamine their assumption of unlimited computing power. As we were finalizing this editorial, Elon Musk’s xAI released Grok-3 chatbot, to rival ChatGPT, DeepSeek, and Llama. Such intense competition will undoubtedly drive rapid advancements and evolutions in generative AI.

Although not as hot as the field of generative AI at this time, the visual sensing and perception domain is still attracting considerable attention. It can benefit from the continuous research and development advances in generative AI. The future of AI-powered visual sensors and sensing holds tremendous promise, with innovations poised to transform industries in numerous fields and beyond.

We have launched the second edition of this Special Issue. This new edition aims to show the latest progress and innovations in AI-powered computer vision for sensor, sensing, and measurement research and applications. It is noteworthy that the scope of the vision iof this Special Issue encompasses a wide range of imaging techniques, including conventional imaging, X-ray imaging, microscopy imaging, magnetic resonance imaging, ultrasound imaging, acoustic imaging, thermal imaging, endoscopic imaging, hyperspectral imaging, radar imaging, and infrared imaging. Our editorial team has been expanded to include co-Guest Editors from a university, a federal research institution, a high-tech company, and a military agency to ensure the diversity of perspectives. We welcome contributions from our readers.

## References

[1] Hinton G.E., Salakhutdinov R.R. (2006). Reducing the Dimensionality of Data with Neural Networks. Science.

[2] Krizhevsky A., Sutskever I., Hinton G.E. ImageNet Classification with Deep Convolutional Neural Networks. Proceedings of the 25th International Conference on Neural Information Processing Systems (NIPS).

[3] Srivastava N., Hinton G., Krizhevsky A., Sutskever I., Salakhutdinov R. (2014). Dropout: A Simple Way to Prevent Neural Networks from Overfitting. J. Mach. Learn. Res..

[4] Cho K., van Merriënboer B., Gulcehre C., Bahdanau D., Bougares F., Schwenk H., Bengio Y. Learning Phrase Representations using RNN Encoder–Decoder for Statistical Machine Translation. Proceedings of the Conference on Empirical Methods in Natural Language Processing (EMNLP).

[5] Sutskever I., Vinyals O., Le Q.V. Sequence to Sequence Learning with Neural Networks. Proceedings of the 28th Conference on Neural Information Processing Systems (NeurIPS).

[6] Goodfellow I., Pouget-Abadie J., Mirza M., Xu B., Warde-Farley D., Ozair S., Courville A., Bengio Y. Generative Adversarial Nets. Proceedings of the Advances in Neural Information Processing Systems (NIPS).

[7] Szegedy C., Liu W., Jia Y., Sermanet P., Reed S., Anguelov D., Erhan D., Vanhoucke V., Rabinovich A. Going Deeper with Convolutions. Proceedings of the IEEE Conference on Computer Vision and Pattern Recognition (CVPR).

[8] He K., Zhang X., Ren S., Sun J. Deep Residual Learning for Image Recognition. Proceedings of the IEEE/CVF Conference on Computer Vision and Pattern Recognition (CVPR).

[9] van den Oord A., Dieleman S., Zen H., Simonyan K., Vinyals O., Graves A., Kalchbrenner N., Senior A., Kavukcuoglu K. WaveNet: A Generative Model for Raw Audio. Proceedings of the 9th ISCA Speech Synthesis Workshop (SSW).

[10] Silver D., Huang A., Maddison C.J., Guez A., Sifre L., van den Driessche G., Schrittwieser J., Antonoglou I., Panneershelvam V., Lanctot M. (2016). Mastering the Game of Go with Deep Neural Networks and Tree Search. Nature.

[11] Vaswani A., Shazeer N., Parmar N., Uszkoreit J., Jones L., Gomez A.N., Kaiser L., Polosukhin I. Attention Is All You Need. Proceedings of the 31st Conference on Neural Information Processing Systems (NeurIPS).

[12] Devlin J., Chang M.W., Lee K., Toutanova K. BERT: Pre-training of Deep Bidirectional Transformers for Language Understanding. Proceedings of the 2019 Conference of the North American Chapter of the Association for Computational Linguistics: Human Language Technologies (NAACL-HLT).

[13] Brown T., Mann B., Ryder N., Subbiah M., Kaplan J.D., Dhariwal P., Neelakantan A., Shyam P., Sastry G., Askell A. Language Models are Few-Shot Learners. Proceedings of the 34th Conference on Neural Information Processing Systems (NeurIPS).

[14] Dosovitskiy A., Beyer L., Kolesnikov A., Weissenborn D., Zhai X., Unterthiner T., Dehghani M., Minderer M., Heigold G., Gelly S. An Image is Worth 16x16 Words: Transformers for Image Recognition at Scale. Proceedings of the International Conference on Learning Representations (ICLR).

[15] Bochkovskiy A., Wang C.Y., Liao H.Y.M. (2020). Yolov4: Optimal speed and accuracy of object detection. arXiv.

[16] Brock A., Donahue J., Simonyan K. (2018). Large Scale GAN Training for High Fidelity Natural Image Synthesis. arXiv.

[17] Karras T., Laine S., Aittala M., Hellsten J., Lehtinen J., Aila T. Analyzing and Improving the Image Quality of StyleGAN. Proceedings of the IEEE/CVF Conference on Computer Vision and Pattern Recognition (CVPR).

[18] Achiam O.J., Adler S., Agarwal S., Ahmad L., Akkaya I., Aleman F.L., Almeida D., Altenschmidt J., Altman S., Anadkat S. (2023). GPT-4 Technical Report. arXiv.

[19] Dubey A., Jauhri A., Pandey A., Kadian A., Al-Dahle A., Letman A., Mathur A., Schelten A., Yang A., Fan A. (2024). The Llama 3 Herd of Models. arXiv.

[20] Team G., Anil R., Borgeaud S., Alayrac J.B., Yu J., Soricut R., Schalkwyk J., Dai A.M., Hauth A., Millican K. (2023). Gemini: A Family of Highly Capable Multimodal Models. arXiv.

[21] Liu Y., Zhang K., Li Y., Yan Z., Gao C., Chen R., Yuan Z., Huang Y., Sun H., Gao J. (2024). Sora: A Review on Background, Technology, Limitations, and Opportunities of Large Vision Models. arXiv.

[22] Yeong D.J., Velasco-Hernandez G., Barry J., Walsh J. (2021). Sensor and Sensor Fusion Technology in Autonomous Vehicles: A Review. Sensors.

[23] Rana K., Khatri N. (2024). Automotive intelligence: Unleashing the potential of AI beyond advanced driver-assistance system, a comprehensive review. Comput. Electr. Eng..

[24] Bajwa J., Munir U., Nori A., Williams B. (2021). Artificial intelligence in healthcare: Transforming the practice of medicine. Future Healthc. J..

[25] Ye M., Ke L., Li S., Tai Y.W., Tang C.K., Danelljan M., Yu F. Cascade-DETR: Delving into High-Quality Universal Object Detection. Proceedings of the IEEE/CVF International Conference on Computer Vision (ICCV).

[26] Kortli Y., Jridi M., Al Falou A., Atri M. (2020). Face Recognition Systems: A Survey. Sensors.

[27] Ma J., He Y., Li F., Han L.J., You C., Wang B. (2024). Segment anything in medical images. Nat. Commun..

[28] Li M., Lv T., Chen J., Cui L., Lu Y., Florencio D., Zhang C., Li Z., Wei F. (2023). TrOCR: Transformer-Based Optical Character Recognition with Pre-trained Models. Proc. AAAI Conf. Artif. Intell..

[29] Xu J., Guo Y., Peng Y. FinePOSE: Fine-Grained Prompt-Driven 3D Human Pose Estimation via Diffusion Models. Proceedings of the 2024 IEEE/CVF Conference on Computer Vision and Pattern Recognition (CVPR).

[30] Nguyen H., Novak E., Wang Z. (2022). Accurate 3D reconstruction via fringe-to-phase network. Measurement.

[31] Mu F., Sifferman C., Jungerman S., Li Y., Han M., Gleicher M., Gupta M., Li Y. Towards 3D Vision with Low-Cost Single-Photon Cameras. Proceedings of the IEEE/CVF Conference on Computer Vision and Pattern Recognition (CVPR).

[32] Zhang H., Ba Y., Yang E., Mehra V., Gella B., Suzuki A., Pfahnl A., Chandrappa C.C., Wong A., Kadambi A. WeatherStream: Light Transport Automation of Single Image Deweathering. Proceedings of the 2023 IEEE/CVF Conference on Computer Vision and Pattern Recognition (CVPR).

[33] Ramazzina A., Bijelic M., Walz S., Sanvito A., Scheuble D., Heide F. ScatterNeRF: Seeing Through Fog with Physically-Based Inverse Neural Rendering. Proceedings of the 2023 IEEE/CVF International Conference on Computer Vision (ICCV).

[34] Muskhelishvili N. (1977). Some Basic Problems of the Mathematical Theory of Elasticity.

[35] Fung Y., Tong P. (2001). Classical and Computational Solid Mechanics.

[36] Altenbach H., Bogdanov V., Grigorenko A., Kushnir R., Nazarenko V., Eremeyev V. (2024). Selected Problems of Solid Mechanics and Solving Methods.

[37] Karaev N., Makarov I., Wang J., Neverova N., Vedaldi A., Rupprecht C. (2024). CoTracker3: Simpler and Better Point Tracking by Pseudo-Labelling Real Videos. arXiv.

[38] Kreyszig E., Kreyszig H., Norminton E.J. (2011). Advanced Engineering Mathematics.

[39] Ciekanowska A., Kiszczak Gliński A., Dziedzic K. (2021). Comparative Analysis of Unity and Unreal Engine Efficiency in Creating Virtual Exhibitions of 3D Scanned Models. J. Comput. Sci. Inst..

[40] Alemohammad S., Casco-Rodriguez J., Luzi L., Humayun A.I., Babaei H., LeJeune D., Siahkoohi A., Baraniuk R.G. Self-Consuming Generative Models Go MAD. Proceedings of the Twelfth International Conference on Learning Representations (ICLR).

[41] Liu Y., Yu L., Wang Z., Pan B. (2023). Neutralizing the impact of heat haze on digital image correlation measurements via deep learning. Opt. Lasers Eng..

[42] Ranftl R., Bochkovskiy A., Koltun V. Vision Transformers for Dense Prediction. Proceedings of the IEEE/CVF International Conference on Computer Vision (ICCV).

[43] Hornik K., Stinchcombe M., White H. (1989). Multilayer Feedforward Networks are Universal Approximators. Neural Netw..

[44] Kidger P., Lyons T. Universal Approximation with Deep Narrow Networks. Proceedings of the 33rd Conference on Learning Theory (COLT).

[45] Pilz K.F., Mahmood Y., Heim L. (2025). AI’s Power Requirements Under Exponential Growth: Extrapolating AI Data Center Power Demand and Assessing Its Potential Impact on U.S. Competitiveness.

[46] Rezvani A., Huang W., Chen H., Ni Y., Imani M. (2024). Self-Trainable and Adaptive Sensor Intelligence for Selective Data Generation. Front. Artif. Intell..

[47] Kanerva P. (2009). Hyperdimensional Computing: An Introduction to Computing in Distributed Representation with High-Dimensional Random Vectors. Cogn. Comput..

[48] Abdulrahman M., Wasif S., Wael M., Azab E., Mashaly M., Abd El Ghany M.A.A. A Review on Hyperdimensional Computing. Proceedings of the 2023 International Conference on Microelectronics (ICM).

[49] Colelough B.C., Regli W. (2025). Neuro-Symbolic AI in 2024: A Systematic Review. arXiv.

[50] Jones N. (2025). How should we test AI for human-level intelligence? OpenAI’s o3 electrifies quest. Nature.

[51] Guo D., Yang D., Zhang H., Song J., Zhang R., Xu R., Zhu Q., Ma S., Wang P., DeepSeek-AI (2025). DeepSeek-R1: Incentivizing Reasoning Capability in LLMs via Reinforcement Learning. arXiv.

